# Observation of glycine zipper and unanticipated occurrence of ambidextrous helices in the crystal structure of a chiral undecapeptide

**DOI:** 10.1186/1472-6807-7-51

**Published:** 2007-08-01

**Authors:** Rudresh Acharya, Madhvi Gupta, Suryanarayanarao Ramakumar, Udupi A Ramagopal, Virander S Chauhan

**Affiliations:** 1 Department of Physics, Indian Institute of Science, Bangalore, India; 2 Bioinformatics Centre, Indian Institute of Science, Bangalore, India; 3 Malaria Lab, International Centre for Genetic Engineering and Biotechnology, New Delhi, India; 4 Department of Biochemistry, Albert Einstein College of Medicine, 1200, Morris Park Avenue, BRONX, New York 10461, USA; 5 Malaria Lab, International Centre for Genetic Engineering and Biotechnology, New Delhi, India

## Abstract

**Background:**

The *de novo *design of peptides and proteins has recently surfaced as an approach for investigating protein structure and function. This approach vitally tests our knowledge of protein folding and function, while also laying the groundwork for the fabrication of proteins with properties not precedented in nature. The success of these studies relies heavily on the ability to design relatively short peptides that can espouse stable secondary structures. To this end, substitution with α, β-dehydroamino acids, especially α, β-dehydrophenylalanine (ΔPhe) comes in use for spawning well-defined structural motifs. Introduction of ΔPhe induces β-bends in small and 3_10_-helices in longer peptide sequences.

**Results:**

The present report is an investigation of the effect of incorporating two glycines in the middle of a ΔPhe containing undecapeptide. A de novo designed undecapeptide, Ac-Gly^1^-Ala^2^-ΔPhe^3^-Leu^4^-Gly^5^-ΔPhe^6^-Leu^7^-Gly^8^-ΔPhe^9^-Ala^10^-Gly^11^-NH_2_, was synthesized and characterized using X-ray diffraction and Circular Dichroism spectroscopic methods. Crystallographic studies suggest that, despite the presence of L-amino acid (L-Ala and L-Leu) residues in the middle of the sequence, the peptide adopts a 3_10_-helical conformation of ambidextrous screw sense, one of them a left-handed (A) and the other a right-handed (B) 3_10_-helix with A and B being antiparallel to each other. However, CD studies reveal that the undecapeptide exclusively adopts a right-handed 3_10_-helical conformation. In the crystal packing, three different interhelical interfaces, Leu-Leu, Gly-Gly and ΔPhe-ΔPhe are observed between the helices A and B. A network of C-H...O hydrogen bonds are observed at ΔPhe-ΔPhe and Gly-Gly interhelical interfaces. An important feature observed is the occurrence of glycine zipper motif at Gly-Gly interface. At this interface, the geometric pattern of interhelical interactions seems to resemble those observed between helices in transmembrane (TM) proteins.

**Conclusion:**

The present design strategy can thus be exploited in future work on de novo design of helical bundles of higher order and compaction utilizing ΔPhe residues along with GXXG motif.

## Background

*De novo *protein design endeavors to construct novel polypeptide sequences that fold into well-defined secondary and tertiary structures resembling those found in native proteins. Many *de novo *design strategies have relied on the known penchants of protein amino acids to espouse various secondary structures leading to several remarkable achievements [1-4]. Alternatively, the amalgamation of conformationally restricted, non-protein amino acids by chemical synthesis has led to triumphant designs of secondary and super secondary structures that mimic proteins [5,6]. In this regard, the ability of α, β-dehydrophenylalanine (ΔPhe) to induce β-bends in small and 3_10_-helices in longer peptide sequences has been well studied [7-18]. The presence of dehydroresidues in peptides confers altered bioactivity as well as increased resistance to enzymatic degradation [19]. Recently designed super secondary structures such as ΔPhe zippers and helical hairpins highlight the potential of ΔPhe to introduce long-range interactions in peptides and it has been noted that the geometry of a 3_10_-helix brings ΔPhe residues at i and i+3 position into a stacking arrangement and the structurally planar ΔPhe side-chains interdigitate to assist the cooperative recognition of helices [5,17,20]. In proteins, there is a wide interplay of weak non-covalent interactions between secondary structural elements, to achieve stability and overall compaction. In this context, in transmembrane proteins it is observed that glycine residues promotes close approach of helices, which permits not only favourable vander Waals interactions of surrounding side chains, but also in many cases, encourage interhelical C^α^-H...O hydrogen bonds [21-25]. Interestingly, it has been found that the GXXXG motif elicits a level of self-association in putative transmembrane helices and the three-residue spacing between both glycines proves to be optimal for the interaction. In an attempt to mimic similar interactions and geometric features, we designed and synthesized an undecapeptide, Ac-Gly^1^-Ala^2^-ΔPhe^3^-Leu^4^-Gly^5^-ΔPhe^6^-Leu^7^-Gly^8^-ΔPhe^9^-Ala^10^-Gly^11^-NH_2. _Its structural features were characterized using X-ray diffraction and Circular Dichroism spectroscopy. ΔPhe residues and glycine residues as GXXG motif were at a two-residue spacer to give rise to a 3_10_-helical conformation. Thus the peptide incorporates two GXXG like motif (Gly^5^-ΔPhe^6^-Leu^7^-Gly^8 ^and Gly^8^-ΔPhe^9 ^Ala^10^-Gly^11^) motif in the helix region and one GXXXG (Gly^1^-Ala^2^-ΔPhe^3^-Leu^4^-Gly^5^) motif near the N-terminus. The bulky leucine residues were placed in middle of the helical segment to ensure that the peptide folds into a right-handed screw sense. A 3_10_-helical conformation of ambidextrous screw sense is established by X-ray diffraction. However, CD studies reveal that a right-handed 3_10_-helical conformation dominate in solution. The preponderance of the right-handed 3_10_-helical conformer is also confirmed using energy calculation studies [Additional file 1]. An unanticipated observation of ambidexterity of the peptide helices in the crystal structure demonstrates the influence of global interactions on the coexistence of left and right-handed helices in the crystal structure. This is a novel observation of a 3_10_-helical dehydroundecapeptide mimicking interhelical interactions as seen amongst transmembrane helices.

## Results and Discussion

### Crystal Structure

**Figure 1:**
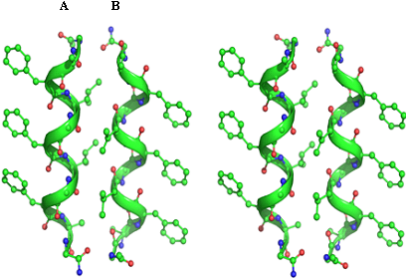
**Stereo view for the molecular conformation of the undeacapeptide**. A denotes a left-handed 3_10_-helix and B aright-handed 3_10 _helix. The helices A and B are antiparallel to each other.

**Figure 2:**
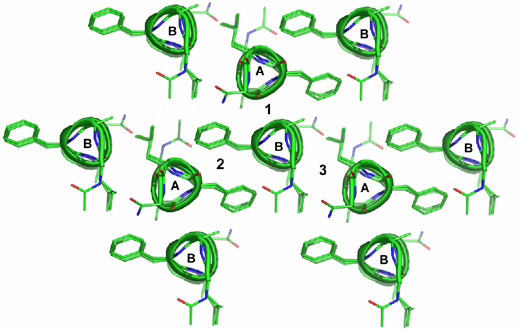
**Arrangement of helices in crystal packing**. The figure shows the arrangement of helices as viewed down the helical axes. There are three interhelical interfaces viz. Gly-Gly (1), ΔPhe-ΔPhe (2) and Leu-Leu (3).

**Figure 3:**
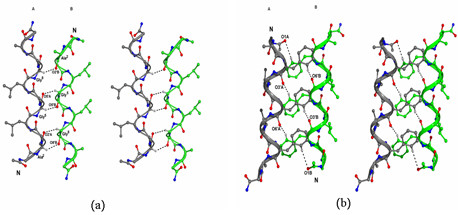
**Network of C-H...O hydrogen bonds at different interfaces**. a) Stereo view for the network of C^α^-H...O hydrogen bonds at Gly-Gly interface. The GXXG motif has promoted the close approach of opposite handed 3_10_-helices there by encouraging the vander Waals and C^α^-H...O interactions. b) Stereo view for the network of C-H...O hydrogen bonds at ΔPhe-ΔPhe interface.

The crystallographic details of the peptide are given in (Table 1). Crystallographic studies suggest that, despite the presence of L-Ala and L-Leu residues in the sequence, the peptide has folded into two conformers in the crystal lattice, conformer A and conformer B (Figure 1). From the main chain conformation angles (Table 2) and the pattern of intramolecular hydrogen bonds (Table 3), it is clear that both right-handed as well as left-handed 3_10_-helices are present in the crystal structure. The average (ϕ,ψ) values for 3_10_- helical stretch (Ala^2^-Ala^10^) in conformer A are (54°, 24°), whereas the average (ϕ,ψ) values for this 3_10_-helical stretch in conformer B are (-59°, -17°). The helices are stabilized by intrahelical 4→1 hydrogen bonds (Table 3). Interestingly the four (L) amino acid residues, Ala^2^, Leu^4^, Leu^5 ^and Ala^10 ^have taken the positive ϕ and ψ values corresponding to the left-handed 3_10_-helical conformation in conformer A (Table 2). In 3_10_-helices, every third residue would lie on the same face of the helix. Consequently the side chains of the three ΔPhe residues in the undecapeptide, ΔPhe^3^, ΔPhe^6^, and ΔPhe^9 ^are stacked on one face of the helix, residues Leu^4^, Leu^7 ^and Ala^10 ^lie on second face of the helix, while Ala^2^, Gly^5 ^and Gly^8 ^lie on third face of the helix. This arrangement of side chains creates a column of protuberant side chains at 120° to each other, resulting in the formation of grooves and wedges. The two helices A and B are antiparallel to each other. The angle between the two helical axes is 179°. It is observed that in crystal lattice the helix A is surrounded by three B helices, similarly helix B is surrounded by three A helices forming ΔPhe-ΔPhe, Leu-Leu and Gly-Gly helical interfaces (Figure 2). The closest approach C^α^-C^α ^distances between the helices A and B at three interfaces was observed to be different; 5.9Å at the ΔPhe-ΔPhe interface, 3.9Å at the Gly-Gly interface and 5.4 Å at the Leu-Leu interface (calculated using computer program Helixang from CCP4 suite). Despite the closest approach of helices at the Gly-Gly interface as compared to Leu-Leu interface, energy calculation studies suggest that the Leu-Leu interface has the maximum stability followed by Gly-Gly and then ΔPhe-ΔPhe interface (Additional file 1). In the crystal lattice, the helices of similar handedness related by translation symmetry are observed as approximate helical rods aligned along z-axis. It is interesting to note that helices of same handedness pack one above the other and stabilize through head-to-tail kind of N-H...O hydrogen bonds; N2...O10', and N12...O1, while the tail to tail hydrogen bonding N12 (A)...O9' (B) is observed between the helices of opposite handedness [26] (Table 4). A notable feature in the crystal structure is that the two shape compliment helices A and B are interacting through extensive network of hydrogen bonds. At the Leu-Leu interface, helices A and B are involved in N-H...O hydrogen bond (Table 4). At the Gly-Gly interface the two conformers A and B are held together by five C^α^-H...O hydrogen bonds all along the helical axis [18]. These backbone (C^α^-H) to backbone (carbonyl) hydrogen bonds observed between C^α^(Ala^2^), C^α^(Gly^5^), and C^α^(Gly^8^) of conformer A to O8', O5' and O2' of conformer B respectively, and conversely C^α^(Gly^5^) and C^α^(Gly^8^) of Conformer B to O5' and O2' of conformer A respectively (Table 4), involve GXXG motifs from the two helices (Fig. 3a, Table 4). At the ΔPhe-ΔPhe interface, helices A and B are held together by symmetrically placed aromatic-backbone C-H...O hydrogen bonds distributed all along the helical axis [18]. Hence C-H (Phenyl)...O (carbonyl) hydrogen bonds are observed between Cδ2 (ΔPhe^3^), Cδ2 (ΔPhe^6^) and Cδ2 (ΔPhe^9^) of conformer A to O6', O3' and O1 of conformer B correspondingly. Similarly C-H (Phenyl)...O (carbonyl) hydrogen bonds are observed between Cδ2 (ΔPhe^3^), Cδ2 (ΔPhe^6^) and Cδ2 (ΔPhe^9^) of conformer B to O6', O3' and O1 of conformer A respectively (Fig. 3b, Table 4). The coexistence of right and left-handed helices favored by the involvement of interhelical hydrogen bonds in the solid state may be presumably to optimize helix-helix interactions, suggesting that tertiary (global) interactions, including overall vander Waals, hydrophobic, electrostatic and hydrogen bond interactions can significantly influence even the local secondary structural features that involves amino acid residues close to each other in a peptide sequence. Glycine residues (Gly^5^, Gly^7^) here seems to act as surrogate D-amino acids by assuming left-handed helical conformation [27]. In particular, the interaction motif which involves the occurrence of aromatic C-H..O hydrogen bonds and intercalation of aromatic side chains between adjacent and antiparallel 3_10_-helices of opposite handedness is observed in other ΔPhe containing peptide crystal structures analyzed earlier in our laboratory [5,17]. It seems that the two opposite handed helices in the crystal packing seen have utilized a similar interaction motif leading to their association with each other. Despite the presence of opposite handed helices, the present peptide is found to engage itself in extensive C-H...O hydrogen bonds. A remarkable feature of the present peptide is the observation of zipper like arrangement of multiple C^α^-H...O hydrogen bonds consistently at three residue intervals at Gly-Gly interface, which may be termed as glycine zipper. The distance of 3.9Å between the adjacent helices at the Gly-Gly interface promotes packing interactions between the helices. This similar geometry for interhelical interaction is reportedly observed in transmembrane helical proteins between helices involving GXXXG like motifs. Although the four-residue spacing is strongly preferred over other possible Gly patterns, reinforcing the significance of the GXXXGXXXG sequence pattern. Nevertheless, other spacings could lead to glycine zipper packing if the Gly residues are placed on the same face of the helix. Thus, the glycine zipper face may act as a magnet for helix packing.

**Table 1:** Data collection and Refinement parameters for Ac-Gly-Ala-ΔPhe-Leu-Gly-ΔPhe-Leu-Gly-ΔPhe-Ala-Gly-NH_2_.

Empirical Formula	C_55 _H_70 _N_12 _O_12_·3H_2_O
Molecular weight	(1091 + 54) Da
Temperature	100 K
Crystal System	Triclinic
Space Group	P1
Cell Parameter	a = 11.2555(6) Å, b = 12.5450(6) Å, c = 21.6444(14) Å α = 75.460(2)°, β = 89.369(2)°,γ = 80.988(5)°
Cell Volume	2920.5(3)Å ^3^
Z (molecules/unit cell)	2
Molecules/asymmetric Unit	2
Density Calculated	1.241 g cm^-3^
μ	8.9 cm^-1^
Radiation used	(λ = 0.92015 Å)
Resolution	0.88 Å
Unique reflections	8082
Observed reflections	7057 [|F_o_| > 4σ(|F_o_|)]
Structure Solution	SHELXS97
Refinement Procedure	Full-matrix least-squares refinement on |F_o_|^2 ^using SHELXL97
Number of parameter refined	1457
Data/Parameter	4.8
R	0.0667 (for [|F_o_| > 4σ(|F_o_|)])
wR2	0.1853 (for all unique reflections)
GooF (s)	1.076
Residual electron density	Max. = 0.41 e/Å ^3^ Min = -0.31 e/Å ^3^

**Table 2:** Torsion angles (°) for peptide.

	Conformer A			Conformer B		
Residue	ϕ	Ψ	ω	ϕ	Ψ	ω
1 GLY	-96	150	171	94	-163	-170
2 ALA	57	30	-174	-71	-5	160
3 ΔPHE	56	13	-170	-52	-18	177
4 LEU	51	25	-175	-63	-13	168
5 GLY	56	19	-171	-55	-21	172
6 ΔPHE	56	19	-172	-52	-21	178
7 LEU	52	28	-177	-65	-13	166
8 GLY	54	23	-172	-57	-20	168
9 ΔPHE	50	23	-175	-50	-21	176
10 ALA	53	38	167	-67	-21	-175
11 GLY	-70	174		75	-168	

**Table 3:** Intrahelical hydrogen bonds observed in the crystal structure of Peptide Ac-Gly-Ala-ΔPhe-Leu-Gly-ΔPhe-Leu-Gly-ΔPhe-Ala-Gly-NH_2_.

Conformer A (left-handed 3_10_-helix)					
Type	Donor (D)	Acceptor (A)	D...A (Å)	H...A (Å)	D-H...A (°)
4→1	N4A	O1'A	2.799	1.96	166
	N5A	O2'A	2.964	2.11	176
	N6A	O3'A	2.836	1.99	167
	N7A	O4'A	2.865	2.02	166
	N8A	O5'A	2.874	2.03	166
	N9A	O6'A	2.868	2.05	158
	N10A	O7'A	2.876	2.03	170

	N11A	O8'A	2.972	2.12	170

Conformer B (right-handed 3_10_-helix)					

Type	Donor (D)	Acceptor (A)	D...A (Å)	H...A (Å)	D-H...A (°)
4→1	N4B	O1'B	2.932	2.09	165
	N5B	O2'B	2.931	2.08	171
	N6B	O3'B	2.872	2.03	168
	N7B	O4'B	2.897	2.04	172
	N8B	O5'B	2.915	2.07	167
	N9B	O6'B	2.802	1.99	157
	N10B	O7'B	2.902	2.05	172

	N11B	O8'B	2.899	2.06	165

**Table 4:** Intermolecular hydrogen bonds observed in the crystal structure of the peptide.

Conformer A						
Type	Donor (D)	Acceptor (A)	D.A (Å)	H...A (Å)	D-H...A (°)	Symmetry
Lateral	N1A	O11'B	2.807	1.99	159	x+1, y-1, z
Leu-Leu interface						
Head-to-tail	N2A	O10'A	2.737	2.09	132	x, y, z+1
	N12A	O1A	3.106	2.26	170	x+1,y, z-1
Tail-to-tail						
	N12A	O9'B	2.877	2.04	165	x+1,y, z-1
Lateral	C3D2A	O6'B	3.224	2.36	154	
ΔPhe-ΔPhe	C6D2A	O3'B	3.260	2.38	158	
Interface	C9D2A	O1B	3.370	2.80	120	
	C2AA	O8'B	3.304	2.60	129	x+1, y, z
Gly-Gly	C5AA	O5'B	3.215	2.54	127	x+1, y, z
Interface	C8AA	O2'B	3.368	2.71	126	x+1,y,z
Solvent	N3A	O1W	2.876	2.03	166	

	C9D2A	O2W	3.426	2.50	176	

Conformer B						

Type	Donor (D)	Acceptor (A)	D.A (Å)	H...A (Å)	D-H...A (°)	Symmetry
Lateral	N1B	O11'A	2.771	1.95	159	x-1, y+1, z
Leu-Leu interface						
Head-to-tail	N2B	O10'B	2.854	2.02	165	x, y, z-1
	N12B	O1B	3.065	2.22	168	x-1, y, z+1
Tail-to-tail						
	N12B	O9'A	3.009	2.19	160	x-1, y, z+1
Lateral	C3D2B	O6'A	3.433	2.57	154	
ΔPhe-ΔPhe	C6D2B	O3'A	3.405	2.53	157	
Interface	C9D2B	O1A	3.508	2.98	118	
Gly-Gly	C5AB	O5'A	3.271	2.66	121	x-1, y, z
Interface	C8AB	O2'A	3.475	2.89	120	x-1, y, z
Solvent	N3B	O2W	2.922	2.08	168	

	C9D2B	O1W	3.417	2.51	166	

### Circular Dichroism studies

**Figure 4:**
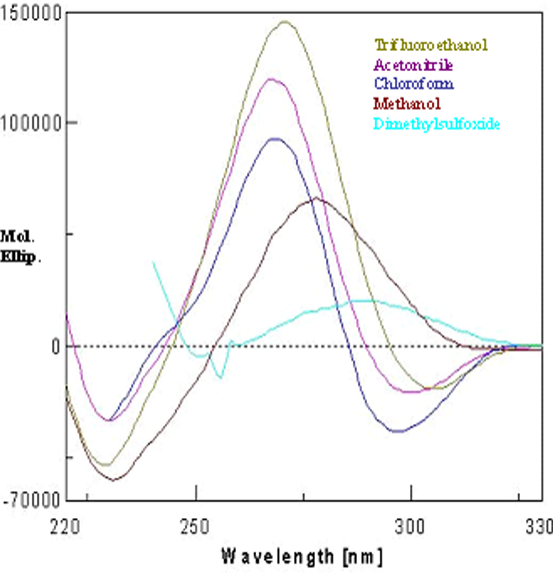
CD spectrum in different solvents.

**Figure 5:**
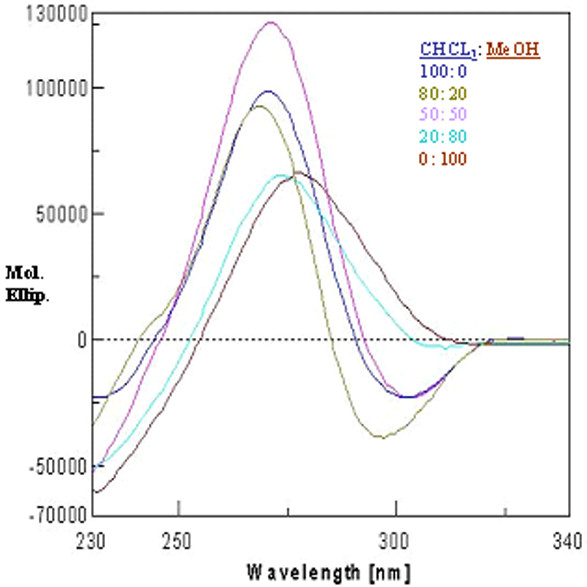
Chloroform-methanol titration depicting maximum intensity at 50: 50 CHCl_3_: MeOH.

**Figure 6:**
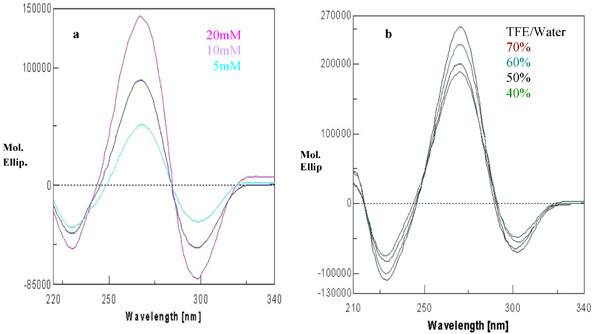
**CD spectra in different lipomimetic solvents**. (a) Different concentrations of SDS-water. (b) Different percentage of aqueous TFE.

**Figure 7:**
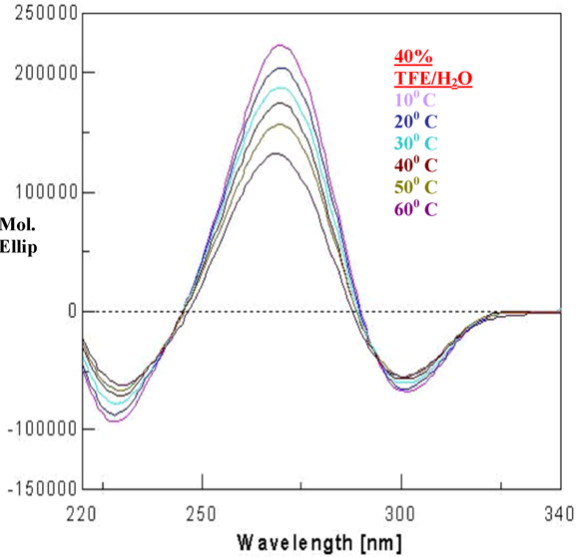
**VT-CD spectrum**. CD spectra in 40% TFE/water as a function of different temperatures.

The peptide has three ΔPhe residues interspersed by two amino acid residues. The CD spectra display a negative couplet (-, +) in acetonitrile, chloroform and trifluoroethanol. A negative band is observed at about 295 nm and an intense positive band at about 265 nm, with a crossover point at ~280 nm (Figure 4). This CD pattern corresponds to the absorption maximum at 270–280 nm and arises from the dipole-dipole interactions between the charge transfer electronic moments of the two dehydroamino acid chromophores placed in a mutual fixed disposition within the molecule. This pattern as reported earlier, is typical of a right-handed 3_10_-helix [13,28]. The varying intensity of bands in different solvents suggests different content of the 3_10_-helical conformer. In methanol, the spectrum shows a positive band at about 280 nm. This could be possible when the styryl side chains of dehydroresidues are placed on the opposite sides of the helix. In this arrangement, no exciton splitting will be observed, and the positive band at 280 nm arises from the contributions of the noninteracting but chirally perturbed chromophores. The very low intensity of bands in the CD spectrum in methanol may be attributed to the polarity of the solvent. It is known that folded peptide structures with stabilizing hydrogen bonds are more stable in apolar solvents than in polar ones. The peptide is found to preferentially form a right-handed 3_10_-helical conformer. The difference between X-ray and CD interpretation may arise due to conformational heterogeneity in the solid state that can lead to crystallization of a minor conformer, driven by favorable packing interactions. On the other hand, the solution studies largely monitor the major species present in solution. The stabilization of right-handed conformer over the left-handed 3_10_-helical conformer is also confirmed using energy calculation studies (Additional file 1). The CHCl_3_-MeOH titrations revealed a surprising but interesting observation. At a concentration of 50:50 (chloroform: methanol), not only the right-handed 3_10_-helical structure is observed but there is also a steep rise in the molar ellipticity value (Figure 5). It is possible that an equal mixture of a polar (methanol) and an apolar (chloroform) solvent provided some kind of amphiphilic environment to the peptide, leading to enhanced stabilization of the structure as compared to that in chloroform alone. Following the above observation, the experiments were performed in different lipomimetic solvents such as aqueous SDS and aqueous TFE mixture. CD spectra of the undecapeptide in SDS and TFE/water solution show intense exciton-coupled band, characteristic of a right-handed 3_10_-helical conformer. Though the peptide was completely insoluble in water but it was soluble in different percentages of SDS/water and TFE/water (Figure 6a). Thus the peptide is found to attain more stability in a membranous environment. The band intensity in TFE/water (40–70%) decreased with the decrease in the percentage of TFE (Figure 6b) and increase in the water content, which is deleterious for dehydrophenylalanine containing structured peptides. However the decrease in band intensity does not reflect in any conformational change of the present peptide even at 40% TFE/water, suggesting the overall stability of the peptide in a membranous environment, provided by TFE/water mixture. Variable temperature studies in 40% TFE/water show maximum stability at 10°C, suggesting the effect of lowering the temperature on the stability of the structure (Figure 7). The explanation for the above observation could be a result of TFE reinforcing hydrogen bonds between carbonyl and amidic NH groups by the removal of water molecules in the proximity of the solute and lowering the dielectric constant of the surrounding milieu [29,30]. Thus the peptide attains more stability in membrane mimetics at relatively low percentage, suggesting the propensity of the peptide to exist in an ordered 3_10_-helical conformation in a hydrophobic environment and depicting stabilization achieved by molecular association [31].

## Conclusion

The present peptide, Ac-Gly-Ala-ΔPhe-Leu-Gly-ΔPhe-Leu-Gly-ΔPhe-Ala-Gly-NH_2, _provides the first example of stability and compaction in interacting helices when glycine residues are incorporated in the middle of the peptide sequence. The incorporation of glycines in the form of GXXG motif along with ΔPhe residue at two-residue spacer has helped in maintaining the 3_10_-helical conformation in both solid as well as solution state. The amalgamation of GxxG motif has not only facilitated the helices to come close at the Gly-Gly interhelical interface but also promoted the formation of glyzine zipper, where a zipper like arrangement of C^α^-H...O hydrogen bonds is observed. The occurrence of weak C-H...O hydrogen bonds at ΔPhe-ΔPhe interface along with occurrence of main chain to main chain C^α^-H...O hydrogen bonds consistently at three residue intervals at Gly-Gly helical interface involving GXXG motifs seems to impart molecular association and stabilization to the interacting helices. The phenomenon of molecular association leading to stabilization of the 3_10_-helical conformer is also confirmed by the solution state study. The present design can encourage the peptide designers in pursuing the ambitious goal of *de novo *design of helical bundles of higher order and compaction utilizing ΔPhe residues along with GXXG motifs.

## Methods

### Peptide synthesis

**Figure 8:**
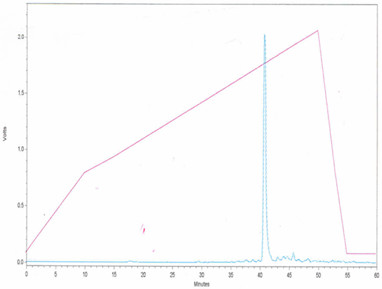
RPHPLC of the peptide.

Fmoc-protected amino acids for solid-phase peptide synthesis were obtained from Novabiochem. The undecapeptide was synthesized manually at a 0.5 mmol scale. Fmoc-Rinkamide MBHA resin (Novabiochem) (0.5 mmol/g) was used to afford carboxyl-terminal primary amide. Couplings were performed by using carbodiimide. The ΔPhe residue was introduced by dehydration of Fmoc-aa-DL-threo-β-Phenyl Serine (AA = glycine or alanine) using fused sodium acetate and freshly distilled acetic anhydride as reported earlier [32]. All reactions were monitored by TLC on precoated silica plates in 9:1 CHCl_3_-MeOH system. The physical characterization of the dipeptide synthons is given as follows: Fmoc-Gly-DL-Phe (β-OH)-OH: Yield = 91.4%, m.p. = 72–74°C, R_f _= 0.40, Fmoc-Gly-ΔPhe-Azlactone: 93%, 102–104°C, 0.95, Fmoc-Ala-DL-Phe (β-OH)-OH: 90%, 112–115°C, 0.3, Fmoc-Ala-ΔPhe-Azlactone: 91%, 142–145°C, 0.7. All the couplings were followed by a five-minute reaction with acetic anhydride and HOBT in DMF/DCM to cap any unreacted amines. Fmoc deprotection was performed with piperidine (20% in DMF). After addition of the final residue, the amino terminus was acetyl-capped and the resin was rinsed with DMF/DCM/MeOH and dried. The final peptide deprotection and cleavage from the resin was achieved with 10 ml of 95:2.5:2.5 TFA: H_2_O: triisopropylsilane for two hours. The crude peptide was precipitated with cold ether, lyophilized and purified by preparative reverse phase HPLC. The crude peptide was purified by RP-HPLC using water-acetonitrile gradient on Waters Deltapak C18 (19 mm × 300 mm). A linear gradient of acetonitrile from 10% to 70% over 60 mins at a flow rate of 6 ml/min was employed. The purified fractions were pooled, lyophilized and stored at -20°C as dry powder. RP-HPLC spectrum of the peptide is given (Figure 8). Retention time: 41.5 mins. Peptide identity was confirmed by mass spectrometer, C_55 _H_70 _N_12 _O_12_, calculated mass 1091da, observed mass 1114 Da (sodium peak), melting point: 160–165°C.

### X-ray crystallography

The peptide crystals were grown by the slow evaporation of peptide solution (1:1 v/v) in ethanol and acetone mixture. The X-ray diffraction data was collected using a suitable crystal cryo cooled to 100 K in synchrotron radiation source, at beam line X9A, Brookhaven National Laboratory. The structure was solved by direct method using SHELXS and was refined using full matrix least square refinement employed in SHELXL [33]. The hydrogen atoms were fixed using stereochemical criteria and were allowed to ride on parent atoms. The crystallographic data of the present peptide is deposited in CCDC (CCDC289231).

### Circular Dichroism studies

CD spectra were recorded on a JASCO J-720 CD spectropolarimeter. The spectra were acquired between 220–330 nm (0.1 cm cell, peptide concentration ~100 μM) at 0.1 nm intervals with a time constant of 4 seconds and a scan speed of 200 nm/min and averaged over 6 separate scans. The spectra obtained were baseline corrected and smoothed. Peptide concentration was determined using the molar extinction coefficient of ΔPhe (~19,000 M^-1^cm^-1^). CHCl_3_-methanol titration was carried out. CD spectra were recorded at different concentrations of SDS and also at different percentage of TFE/water. The CD spectra were recorded in 40% TFE/water at variable temperatures.

### Energy calculation

The energy minimization for the present peptide was performed using the SYBYL software package (version 7.0) (1). The force field used was AMBER7 FF99 implemented in SYBYL. The convergence criterion of 0.05 kcal/mol (Å) as well as the non-bonded cut-off distance was set to 8Å. The partial charges on protein residues were AMBER7 F99 all-atom charges. A value of 1 was set out for dielectric constant for these peptides. The details of energy calculation values are given as additional file 2.

## Abbreviations

Ac: Acetyl

CHCl_3_: Chloroform

DCM: Dichloromethane

DMF: N, N-Dimethylformamide

Fmoc: 9-Fluorenylmethoxycarbonyl

Rinkamide MBHA resin: 4-(2',4'-Dimethoxyphenyl-Fmoc-aminomethyl)-phenoxyacetamido-norleucyl-methylbenzhydrylamine resin

H_2_O: Water

MeOH: Methanol

SDS: Sodium dodecyl sulphate

TFA: Trifluoroacetic acid

TFE: Trifluoroethanol

TLC: Thin layer chromatography

## Authors' contributions

RA solved the crystal structure of the peptide, carried out energy calculation studies, analysis and interpretation of crystal data. MG carried out the peptide synthesis, purification and characterization, acquired the CD spectra and performed the analysis of the CD data. UAR collected and processed the synchrotron diffraction data for the crystal. RA, MG, SR, and VSC conceived of the study, and participated in its design and coordination and helped to draft the manuscript. All authors read and approved the final manuscript.

## Supplementary Material

Additional file 1 Energy Calculation Studies. Energy values at various interfaces, calculated using software SYBYL. Click here for file

Additional file 2 Energy Calculation Studies. Click here for file
