# Supplementary material for: Observation of glycine zipper and unanticipated occurrence of ambidextrous helices in the crystal structure of a chiral undecapeptide
Source: BMC Struct Biol. 2007 Aug 1;7:51. doi: 10.1186/1472-6807-7-51 (PMC2042501; doi:10.1186/1472-6807-7-51)
Supplement: Additional file 1 — Energy Calculation Studies. Energy values at various interfaces, calculated using software SYBYL. [file 1472-6807-7-51-S1.doc]

**Energy Calculation Studies**

Energy values at various interfaces, Leu-Leu interface more stable than others. Gly-Gly interface is more stable than Phe-Phe interface. Energy values are calculated using software SYBYL.

**Energy for Leu-Leu interface:**

Bond Stretching Energy: 6.892

Angle Bending Energy: 20.946

Torsional Energy: 35.369

Improper Torsional Energy: 1.275

1-4 vander Waals Energy: 75.941

Vander Waals Energy: -79.891

1-4 Electrostatic Energy: 251.892

Electrostatic Energy: -579.460

=========================

Total Energy: -267.036 kcals/mol

=========================

**Energy for Gly-Gly interface:**

Bond Stretching Energy: 6.720

Angle Bending Energy: 20.791

Torsional Energy: 36.499

Improper Torsional Energy: 1.463

1-4 vander Waals Energy: 76.790

vander Waals Energy: -86.020

1-4 Electrostatic Energy: 252.278

Electrostatic Energy: -573.747

=========================

Total Energy: -265.226 kcals/mol

=========================

**Energy for ΔPhe-ΔPhe interface:**

Bond Stretching Energy: 6.326

Angle Bending Energy: 21.505

Torsional Energy: 35.417

Improper Torsional Energy: 1.324

1-4 van der Waals Energy: 76.348

van der Waals Energy: -90.324

1-4 Electrostatic Energy: 252.529

Electrostatic Energy: -565.047

=========================

Total Energy: -261.921 kcals/mol

=========================

**Energy values, calculated for conformer A and B:**

**Energy for conformer A:**

Bond Stretching Energy: 3.798

Angle Bending Energy: 10.768

Torsional Energy: 18.495

Improper Torsional Energy: 0.446

1-4 van der Waals Energy: 38.763

vander Waals Energy: -25.479

1-4 Electrostatic Energy: 126.673

Electrostatic Energy: -280.305

=========================

Total Energy: -106.842 kcals/mol

=========================

**Energy for conformer B:**

Bond Stretching Energy: 3.213

Angle Bending Energy: 8.835

Torsional Energy: 16.278

Improper Torsional Energy: 0.713

1-4 van der Waals Energy: 37.762

van der Waals Energy: -28.436

1-4 Electrostatic Energy: 125.623

Electrostatic Energy: -279.822

=========================

Total Energy: -115.833 kcals/mol

=========================

Conformer A (left-handed helix) is less stable than conformer B (right-handed helix).
