# Supplementary material for: Observation of glycine zipper and unanticipated occurrence of ambidextrous helices in the crystal structure of a chiral undecapeptide
Source: BMC Struct Biol. 2007 Aug 1;7:51. doi: 10.1186/1472-6807-7-51 (PMC2042501; doi:10.1186/1472-6807-7-51)
Supplement: Additional file 2 — Energy Calculation Studies. [file 1472-6807-7-51-S2.doc]

Additional file 2

File format: DOC

Title: Energy Calculation Studies.

Description: Energy values, calculated for conformer A and B.

**Energy for conformer A:**

Bond Stretching Energy: 3.798

Angle Bending Energy: 10.768

Torsional Energy: 18.495

Improper Torsional Energy: 0.446

1-4 van der Waals Energy: 38.763

vander Waals Energy: -25.479

1-4 Electrostatic Energy: 126.673

Electrostatic Energy: -280.305

=========================

Total Energy: -106.842 kcals/mol

=========================

**Energy for conformer B:**

Bond Stretching Energy: 3.213

Angle Bending Energy: 8.835

Torsional Energy: 16.278

Improper Torsional Energy: 0.713

1-4 van der Waals Energy: 37.762

van der Waals Energy: -28.436

1-4 Electrostatic Energy: 125.623

Electrostatic Energy: -279.822

=========================

Total Energy: -115.833 kcals/mol

=========================

Conformer A (left-handed helix) is less stable than conformer B (right-handed helix).
